# Isolated duodenal ischemia of unknown etiology: a case report

**DOI:** 10.1186/s12893-021-01425-7

**Published:** 2021-12-18

**Authors:** Elahe Meftah, Narjes Mohammadzadeh, Faeze Salahshour

**Affiliations:** 1 Students’ Scientific Research Center, Tehran University of Medical Sciences, Tehran, Iran; 2 Department of Surgery, Imam Khomeini Hospital Complex, Tehran University of Medical Sciences, Tehran, Iran; 3 Department of Radiology, Advanced Diagnostic and Interventional Radiology Research Center (ADIR), Tehran University of Medical Sciences, Tehran, Iran; 4 Liver Transplantation Research Center, Imam-Khomeini Hospital, Tehran University of Medical Sciences (TUMS), Tehran, Iran

**Keywords:** Duodenal ischemia, Duodenal necrosis, Myelodysplastic syndrome, Case report

## Abstract

**Background:**

Acute abdomen is among the most common presentations observed in clinical practice. The present study describes a patient with isolated duodenal ischemia as an extremely rare etiology of acute abdomen.

**Case presentation:**

A 79-year-old male with acute abdominal pain, nausea, and vomiting presented to the emergency department of our hospital. He was diagnosed with myelodysplastic syndrome 7 years ago, for which he took thalidomide and erythropoietin as the main medications. The prominent findings of the physical examination were hypotension, tachycardia, fever, mild hypoxemia, and epigastric and right upper quadrant tenderness of the abdomen. Except for mildly increased creatinine and lipase, other laboratory findings were in concordance with myelodysplastic syndrome. Due to the patient’s oliguria, the computed tomography (CT) scan was performed without contrast, which, together with the ultrasonography, raised the clinical impression of acute pancreatitis. The patient’s hypotension was refractive to supportive treatment, resulting in progressive deterioration of the clinical condition. A later contrast-enhanced CT scan suggested microvascular ischemia of the duodenum. An emergent Whipple’s procedure was planned initially, which was later switched to a damage control surgery due to the patient’s cardiac arrest during the surgery. Despite all the supportive therapy provided at the intensive care unit, the patient expired of a cardiac arrest which occurred two hours after the termination of the surgery.

**Conclusions:**

The high rate of mortality in duodenal necrosis necessitates emergent diagnosis and proper management. When other common etiologies are ruled out, clinicians should consider duodenal pathology as a potential cause of acute abdomen.

## Background

Duodenal ischemia is one of the most challenging and rarest surgical conditions. This condition may manifest with acute abdomen, one of the most common presentations observed in clinical practice. The literature on this subject is scant and mainly limited to a few published case reports [1–5]. Here we describe an elderly patient with duodenal necrosis of unknown origin who presented to the emergency ward of a tertiary referral hospital.

## Case presentation

The patient was a 79-year-old Caucasian male with a 7-year history of myelodysplastic syndrome (MDS) and a complaint of abdominal pain. The pain started in the evening of the day before and was accompanied by fever, oral intolerance, nausea, and vomiting. He did not complain of bowel habit changes, although he had a history of chronic constipation. Past medical and surgical history was positive for MDS and cholecystectomy. MDS was controlled with daily thalidomide, deferasirox, dimethicone, pantoprazole, gabapentin, vitamin B12, and folic acid. He also took erythropoietin and rivaroxaban three times a week and filgrastim every fifth day. The family history and habitual history of the patient were unremarkable.

The patient was awake and oriented on admission, yet he was ill, dehydrated, and mildly agitated. He had blood pressure 95/60 mmHg, pulse rate 105/min, respiratory rate 17/min, Temperature 37.9 °C, and O_2_ Saturation 90% on ambient air. Tenderness of the epigastrium and right upper quadrant of the abdomen was noted, without abdominal distension, rebound tenderness, or guarding. Laboratory results were as mentioned in Table 1. The patient’s electrocardiogram was unremarkable.

**Table 1:** Laboratory results of the patient

Test	Result	Unit	Reference interval
WBC	2.8	×10^3^/mm^3^	4.1–10.1
RBC	2.18	×10^6^/mm^3^	4.2–5.8
Hemoglobin	6.6	g/dL	12–16
Hematocrit	21.1	%	36–51
Platelet	252	×10^3^/mm^3^	150–400
Na	139	meq/L	135–145
K	3.7	meq/L	3.5–5.0
Blood glucose	163	mg/dL	–
Urea	87	mg/dL	15–50
Creatinine	1.8	mg/dL	0.5–1.0
CRP	47	mg/L	< 6.0
PT	16.9	s	11–15
Control	12.2	s	–
INR	1.38	s	1–1.4
PTT	32	s	25–40
AST	42	U/L	< 37
ALT	45	U/L	< 41
ALP	133	U/L	70–306
LDH	483	U/L	< 480
Amylase	50	U/L	< 100
Lipase	96	U/L	≤ 60

Plain thoracic and abdominopelvic radiographs were normal. In abdominopelvic ultrasonography, mild fluid in subhepatic and inflamed echogenic fat in the upper abdomen and around the pancreas was found, along with duodenal wall thickening. With the impression of pancreatitis, intravenous Ciprofloxacin, Metronidazole, Ondansetron, normal saline, and one unit of packed red blood cells were administered. As the patient did not have a proper urinary output (200ml since urinary catheterization), the computed tomography (CT) scan was performed without intravenous contrast. The abdominopelvic CT scan demonstrated edematous wall thickening of the entire duodenum with water halo and significant adjacent fat stranding and swelling of the pancreas. Pancreatitis was a potential etiology of secondary duodenal wall thickening [5]. However, observing the bulk of the pathologic changes at the duodenum and severe duodenal wall edema also raised the possibility of primary duodenal pathology. Accordingly, a sign of ischemia was suggested, and a contrast-enhanced CT scan was advised to evaluate the related vessels further.

**Fig. 1:**
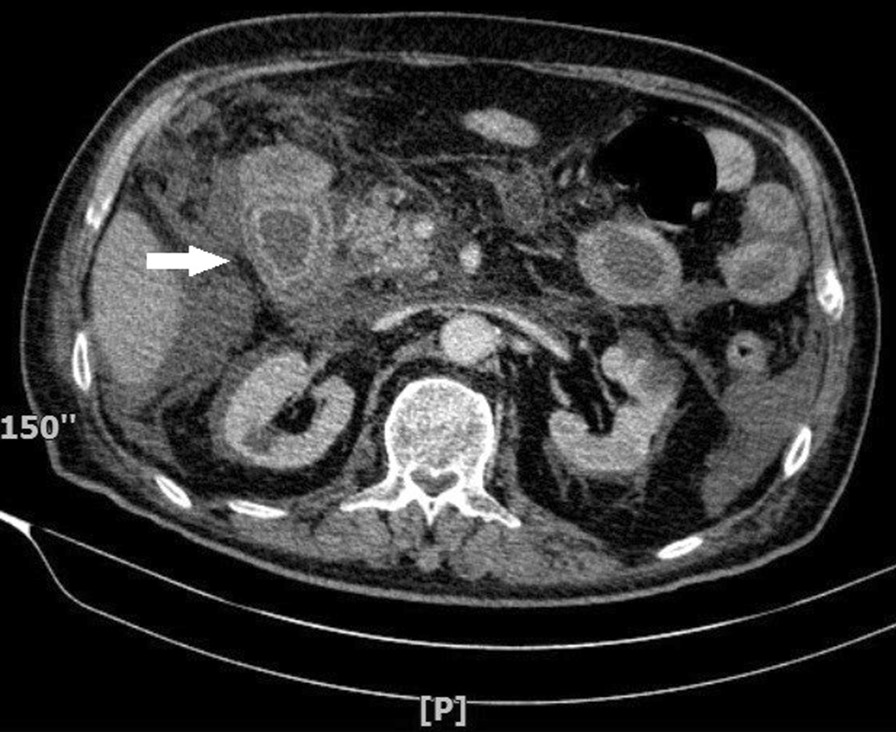
Contrast-enhanced CT scan in favor of ischemic duodenum (white arrow)

The patient remained hypotensive and oliguric regardless of intravenous fluid resuscitation. Nine hours after admission, norepinephrine was initiated. However, blood pressure remained low and fluctuated during the next five hours, with the minimum being 60/40 mmHg. Despite supportive treatment, the patient’s condition deteriorated to the extent of severe electrolyte imbalance, gasping, severe acidosis (arterial pH = 6.8), and GCS 4/15. Due to the mentioned deterioration, another abdominopelvic CT scan with intravenous contrast was requested at the fourteenth hour of admission. The second CT scan demonstrated a marked decrease in the enhancement of the edematous duodenum and target sign. Since the related visible vessels were patent, microvascular ischemia or necrosis of the duodenum was highly suggested (Fig. 1).

**Fig. 2:**
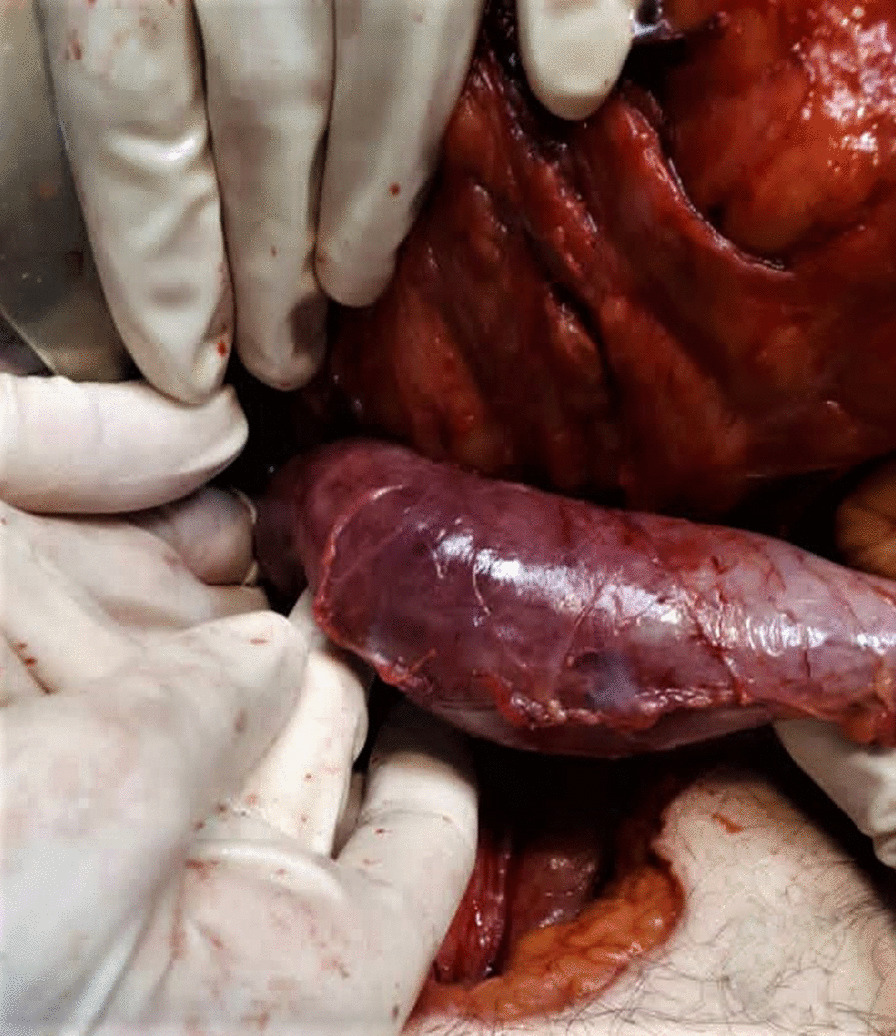
Surgical exploration revealing ischemia of duodenum

**Fig. 3:**
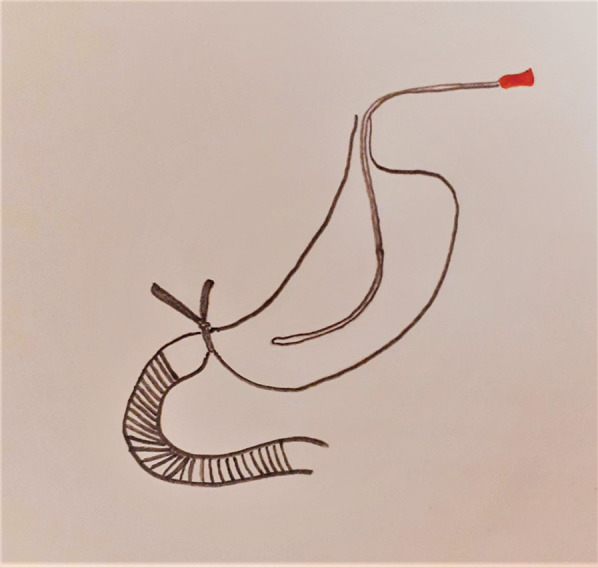
Schematic picture of pyloric exclusion technique with polyester tape

The patient was immediately transferred to the operating room in a critical condition. With a midline laparotomy incision, 400 mL serous fluid was drained. Kocher’s maneuver was performed to explore the duodenum and the retroperitoneal structures. Duodenum (D2–D3–D4) was grossly ischemic, the stomach was dilated, and the small bowel distal to the Treitz ligament appeared normal (Fig. 2). The Pancreas was normal in view, without any saponification, inflammation, or edema in favor of pancreatitis. Exploration of the superior mesenteric artery and vein did not reveal any pathologic findings. Whipple’s procedure was planned initially. However, the patient’s cardiac arrest during the operation directed the plan to a damage control surgery. The overall operation time was approximately one hour and a half. To do the pyloric exclusion in the shortest time, the pylorus was isolated, and a polyester surgical tape was passed behind that. Tight ligation of the tape around the pylorus temporarily excluded the ischemic duodenum. To achieve a damage control surgery, decompression of the stomach was done by a red (18 French) nasogastric tube (Fig. 3). Lastly, an open (corrugated sheet) drain was placed near the duodenum, and the patient was then transferred to the intensive care unit. Despite two hours of supportive therapy, blood pressure remained 40 mmHg/pulse, leading to a cardiac arrest with asystole rhythm and patient expiration. According to the refusal of next of kin to consent, an autopsy was not performed.

## Discussion

Duodenal necrosis is a rare but lethal condition, with an almost 40% mortality rate [1]. With its rich blood supply, the incidence of isolated duodenal necrosis remains an enigma. The previously-explained etiologies of duodenal ischemia include pancreatitis [5, 6], vasculitis [2], caustic injury, trauma, Jejunal intussusception [1] or high loop obstruction [3], and perinephric abscess [7]. Nevertheless, the etiology remains unknown in some patients [4, 6], similar to the present case. There was no evidence of caustic injury or trauma in the history and physical examination of the patient. Additionally, the CT scan and surgical exploration ruled out Jejunal obstruction, pancreatitis, perinephric abscess, and pathologies of the large vessels. Here we hypothesize other possible etiologies of duodenal ischemia in the present case.

The present case was complicated with myelodysplastic syndrome (MDS), a disorder with ineffective hematopoiesis and occasional coagulopathy. The MDS treatment regimen and the course of the disease might have contributed to duodenal ischemia in the present case.

With its anti-angiogenic potential, thalidomide could be suspected as an etiology of duodenal ischemia. Thromboembolic events (TEEs) are among the rare complications of thalidomide monotherapy. Occasional cases of TEEs with immunomodulatory drugs combined with anticoagulants are also reported [8–10]. As thalidomide and lenalidomide show almost similar rates of TEEs [11], we reviewed the literature for TEEs associated with any of the two mentioned immunomodulatory drugs (IMiDs). Deep vein thrombosis, pulmonary thromboembolism, myocardial infarction, and cerebrovascular accident are the expected forms of TEEs associated with IMiDs [8, 9, 11]. However, some unusual TEEs, including concurrent cerebrovascular accident and critical upper limb ischemia [12] and superior vena cava thrombosis [8] are also reported in the case reports of myeloma patients treated with thalidomide. Except for concomitant corticosteroid consumption in some of the mentioned cases [9, 12], no other risk factors for thrombosis were present. Whether the combination therapy of thalidomide and erythropoietin for the present case has been an additional risk of thrombosis is still controversial. Post-marketing analyses suggest an increased rate of TEEs in the combination therapy compared to the monotherapy [13]. However, the clinical trials do not confirm a significant difference [10, 14].

Vasculitis is another possible etiology for duodenal ischemia [2]. The prevalence of vasculitis in the MDS population is estimated at approximately 20% [15]. MDS-associated vasculitis may not demonstrate the typical symptoms [15, 16] and worsens MDS prognosis [15, 17]. Unusual TEEs have been observed in the setting of MDS in association with vasculitis. Incalzi et al. describe a case of central nervous system vasculitis in a patient with MDS, leading to diffuse ischemic damage of the central nervous system [18]. When common etiologies are ruled out, other ischemia of unknown etiology in patients with MDS (e.g., uterine [19] or splenic infarction [20]) could also be attributed to vasculitis [21–23]. The present case did not have symptoms or signs in favor of systemic vasculitis or involvement of any large abdominal vessels. However, isolated gastrointestinal vasculitis with medium or small vessel involvement is still a possible etiology of bowel ischemia in this patient. Segmental bowel ischemia is specifically expected when small vessels are involved. The challenging diagnosis and management of isolated gastrointestinal vasculitis pose the necessity of a medico-surgical interdisciplinary approach. As acute abdomen is the most common presentation of isolated gastrointestinal vasculitis, surgical resection and pathologic evaluation are the diagnostic and curative approaches for these cases. Immunosuppressive agents are the treatment of choice when the diagnosis is ascertainable before surgical intervention [24].

Whatever the etiology, duodenal ischemia requires emergent diagnosis and appropriate treatment. The CT scan findings in favor of duodenal necrosis include severe duodenal wall thickening and edema with water density of submucosa, which together create a target sign. Markedly reduced duodenal wall enhancement and significant stranding of the surrounding fatty tissue are other CT scan findings. Intestinal pneumatosis may also be visible in the advanced stages and is associated with an adverse prognosis [25]. When duodenal necrosis or peritonitis are suspected, surgical intervention may be mandatory. The surgical approach is dependent upon the site and size of the ischemia. The isolated ischemia or necrosis of D1 needs resection of the damaged part, and also end-to-end anastomosis is acceptable. The involvement of D2 is a complicated feature that needs Whipple procedure or near-total duodenectomy [3]. The necrosis or ischemia of D3 or D4 can be managed by resecting the pathologic part and reconstruction with Roux-en-Y duodenojejunostomy or end-to-end duodenojejunal anastomosis [26].

Multidisciplinary discussion between the radiologist and the surgeon was among the strengths of this case management. There were also some limitations in the management of the present case. The rapid deterioration of the patient’s condition limited the utilization of further tests to reveal the etiology of duodenal necrosis. There was also a delay in the diagnosis, mainly due to the patient’s oliguria and the subsequent delay of contrast-enhanced CT scanning.

In conclusion, duodenal necrosis is a rare, fatal, and rapidly progressive condition that requires emergent intervention. Diagnosis is possible based on the CT scan findings, including significant reduction of the duodenal wall enhancement and the presence of duodenal wall edema and target sign. Surgery may be favored based on the patient’s clinical condition, with its approach chosen according to the site of involvement.

## Data Availability

All the relevant data have been provided in this published article.

## Abbreviations

MDS: Myelodysplastic syndrome
CT: Computed tomography
TEEs: Thromboembolic events
IMiDs: Immunomodulatory drugs
